# High-Performance Passive Plasma Separation on OSTE Pillar Forest

**DOI:** 10.3390/bios11100355

**Published:** 2021-09-25

**Authors:** Zhiqing Xiao, Lexin Sun, Yuqian Yang, Zitao Feng, Sihan Dai, Hao Yang, Xingwei Zhang, Chia-Lin Sheu, Weijin Guo

**Affiliations:** 1 Department of Biomedical Engineering, Shantou Univeristy, Shantou 515063, China; 19zqxiao@stu.edu.cn (Z.X.); 19lxsun@stu.edu.cn (L.S.); 18yqyang3@stu.edu.cn (Y.Y.); 20ztfeng@stu.edu.cn (Z.F.); xujialin@stu.edu.cn (C.-L.S.); 2 Department of Biology, Shantou Univeristy, Shantou 515063, China; 20shdai@stu.edu.cn; 3 Department of Physics, Shantou Univeristy, Shantou 515063, China; 19hyang@stu.edu.cn; 4 Department of Mechatronic Engineering, Shantou Univeristy, Shantou 515063, China; zhangxw@stu.edu.cn

**Keywords:** plasma separation, OSTE, pillar forest, filtration membrane, separation yield, protein recovery rate, lateral flow tests

## Abstract

Plasma separation is of high interest for lateral flow tests using whole blood as sample liquids. Here, we built a passive microfluidic device for plasma separation with high performance. This device was made by blood filtration membrane and off-stoichiometry thiol–ene (OSTE) pillar forest. OSTE pillar forest was fabricated by double replica moldings of a laser-cut polymethylmethacrylate (PMMA) mold, which has a uniform microstructure. This device utilized a filtration membrane to separate plasma from whole blood samples and used hydrophilic OSTE pillar forest as the capillary pump to propel the plasma. The device can be used to separate blood plasma with high purity for later use in lateral flow tests. The device can process 45 μL of whole blood in 72 s and achieves a plasma separation yield as high as 60.0%. The protein recovery rate of separated plasma is 85.5%, which is on par with state-of-the-art technologies. This device can be further developed into lateral flow tests for biomarker detection in whole blood.

## 1. Introduction

Blood contains many biomarkers that are related to the health conditions of human body, from indexes of nutrients (such as glucose and vitamin) to cancer markers (such as prostate-specific antigen) [1,2,3]. Therefore, many point-of-care diagnostic platforms use whole blood as sample liquids for testing. Plasma separation is needed for many tests to avoid the disturbance of hemoglobin from red blood cells. Active and passive methods are used to filter blood cells from whole blood using microfluidic systems [4]. An external power source is needed for active plasma separation, such as pressure source and electrical field. Tripathi et al. built a device that utilized biophysical and geometrical effects to filter plasma in a continuous flow fashion [5]. A standalone 3D-printed microfluidic device was developed to filter red blood cells using the microfiltration method [6]. Inertial microfluidics can be used to separate plasma from whole blood sample in a high-throughput way [7,8]. Blood separation can also be achieved on microfluidic devices using acoustophoresis [9] and dielectrophoretic force [10]. Household tools or toys including egg beaters [11], smartphones [12], and paper centrifuges [13] have been developed to separate plasma from blood. To fit the application circumstance of point-of-care diagnostics including lateral flow tests, passive methods are often chosen to separate plasma from whole blood sample. Maria et al. has developed a microfluidic device using the sedimentation effect of red blood cells to separate plasma [14]. Plasma filtration membrane is used together with capillary channels to achieve hemolysis-free plasma separation [15]. Shamsi et al. utilized the Zweifach–Fung effect and collected plasma in flow branches with a higher flow rate [16]. Guo et al. built a blood filter using OSTE synthetic paper, which induced the local agglutination of red blood cells by precoating agglutination antibodies on its surface [17]. Baillargeon et al. made a paper microfluidic device by integrating a prefilter, a plasma separation membrane, and absorbent material to yield a high separation efficiency [18]. According to the working principle, passive plasma separation methods including sedimentation, filtration, and hydrodynamic effects have been reviewed in detail in Reference [19]. For lateral flow tests using whole blood as sample liquids, usually, there are two options for plasma separation: pretreatment by centrifuge and microfiltration by filtration membrane. By integrating a filtration membrane on the test strip, lateral flow tests can separate plasma on site and use the plasma for later analyses [20]. For traditional lateral flow tests working on blood, the filtration membrane is laminated on the lateral flow test substrate: nitrocellulose paper, which acts as a capillary pump to propel the separated plasma. However, nitrocellulose paper has some disadvantages as a lateral flow test substrate, including nonuniform microstructures and strong autofluorescence. In this work, we developed a new substrate material for such applications: OSTE pillar forest. Pillar forest has been extensively used as a substrate for capillary flow [21,22,23,24]. Due to the uniform capillary flow rates provided by pillar forest, it is often used as a lateral flow test substrate [25,26,27], which can help to generate uniform signals and reduce the variation of testing results. OSTE is a photocurable polymer that was developed for biochip fabrication [28,29,30]. OSTE pillar forest was fabricated by double replica moldings of a laser-cut PMMA mold, which has a uniform microstructure. Moreover, the polymer OSTE has easily tuned surface properties and low autofluorescence [31]. With free thiol groups, it is easy to immobilize proteins on OSTE surface by click chemistry, such as the protocols thiol–yne and thiol–maleimide [32,33]. Synthetic paper fabricated by OSTE has been used as a substrate for protein microarray, which couples fluorescent immunoassays on its surface via a thiol–maleimide reaction [31]. Compared with nitrocellulose and glass, synthetic paper can improve the performance of microarray immunoassays [31]. The characteristics of OSTE pillar forest can make up for the shortcomings of nitrocellulose. We built the passive microfluidic device by gluing a filtration membrane onto the OSTE pillar forest substrate, and the other surface of OSTE pillar forest was covered by a hydrophilic tape to enhance the capillary action. High-performance plasma separation can be achieved on this passive device. We tested blood samples with different hematocrit values, calculated the plasma separation yield, and checked the purity of separated plasma. In addition, we analyzed the variation of sample flow rates among different devices and different blood samples. Moreover, we calculated the protein recovery rate of the separated plasma and purification efficiency of our devices.

## 2. Materials and Methods

The blood filtration membrane was purchased from Cobetter (PSM0180-B, Hangzhou, China). Polydimethylsiloxane (PDMS, Sylgard 184) was from Dow Corning Corporation (Midland, MI, USA) and prepared by mixing base and curing agent in a weight ratio as 10:1. The hydrophilic tape (ARflow^®^ 93049) was provided by Adhesives Research (Shanghai, China). The free nail glue was purchased from Feifanli (Shenzhen, China). The whole blood samples were from adult pigs provided by a local food provider. The blood samples were immediately stored in a blood collection tube (BD Vacutainer^®^, K2 EDTA 10.8 mg, 6 mL, REF: 367863, Franklin Lakes, NJ, USA) after they were collected from pigs, and then stored at 4 ${}^{\circ }$C. All of the experiments were conducted within three days after the collection of blood samples. We measured the hematocrit of blood samples by centrifuging EDTA-treated whole blood in a capillary tube.

**Figure 1:**
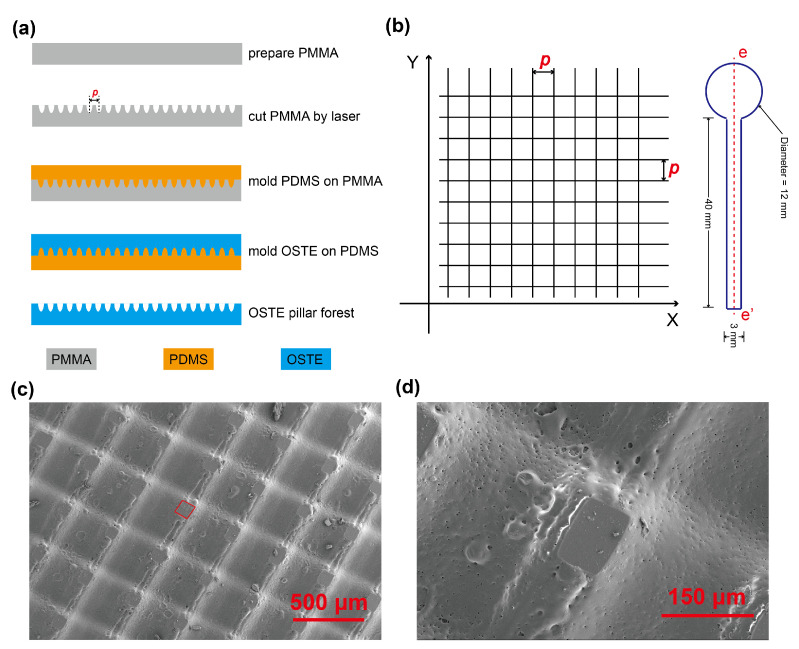
Fabrication and design of OSTE pillar forest. (**a**) The fabrication procedures of OSTE pillar forest: at first, we engraved the PMMA using a laser; then, we used the PMMA to mold PDMS; and after that, we used the PDMS negative to mold OSTE. (**b**) The cutting pattern of the laser on PMMA (left) and the design of the OSTE pillar forest strip (right). The main axis (e-e’) of the OSTE pillar forest strip is along the Y axis. (**c**,**d**) SEM pictures of OSTE pillar forest.

**Figure 2:**
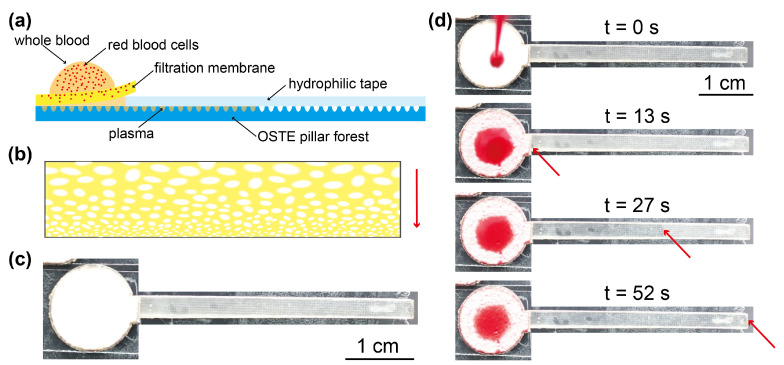
Design of the passive microfluidic device and experiments of plasma separation on the device. (**a**) The schematic of cross section of the device. Part of the OSTE pillar forest substrate is covered by the hydrophilic tape, and part of the OSTE pillar forest substrate is covered by the filtration membrane. There is a void region with a wedge shape between the filtration membrane, hydrophilic tape, and OSTE pillar forest. (**b**) The microstructure of filtration membrane (not to scale). The pore size on one side of the membrane is bigger than that on the other side (contacting OSTE pillar forest substrate); the red arrow indicates the direction of blood flow. (**c**) A real device. (**d**) The whole process of plasma separation on a device: we loaded 45 μL of whole blood on the center of the filtration membrane; then, we found that plasma appeared in the region under the hydrophilic tape. After that, plasma continued pumping out until the OSTE pillar forest substrate was filled with plasma. The red arrows indicate the position of the plasma front.

For the fabrication of OSTE pillar forest (as shown in Figure 1a), at first, we used a laser cutter (Model: Leidayu, Thunderlaser, Dongguan, China) to engrave the PMMA plate and form microgrooves on the PMMA surface. The parameters of PMMA engraving were power, 25%; speed, 100 mm/s; laser frequency, 20 KHz; and number of passes, 1. The cutting pattern on the PMMA plate was orthogonal lines, shown in Figure 1b. At first, we cut the lines along the X axis and then the lines along the Y axis. The long axis of the OSTE pillar forest strip in this study was along the Y axis. We tested two pitch distances (*p* in Figure 1a,b): 350 μm and 500 μm. After we prepared the PMMA mold, we poured PDMS on the PMMA and cured PDMS. After that, we removed the PDMS negative from the PMMA mold and used it as a mold to make an OSTE replica [34], of which the details can be found in Appendix A.1. The curing of the polymer was performed by flood ultraviolet (UV) irradiation (UV radiation lamp, Asiga, Australia). Then, we removed the OSTE from PDMS and obtained a piece of OSTE pillar forest substrate, of which the average thickness was 944 μm. We measured and observed the microstructures of OSTE pillar forest by step profiler (Dektak XT, Bruker, Billerica, MA, USA) and SEM (Gemini 300, Zeiss, Germany). Then, we cut the OSTE pillar forest piece as well as the filtration membrane piece to the desired shapes using the laser cutter. The shape design was by the software LaserMaker (Thunderlaser, Dongguan, China). The shape of the filtration membrane was a circle with a diameter of 12 mm and a rectangle with the dimension 2 × 3 mm, and the shape of OSTE pillar forest was a circle with a diameter of 12 mm and a rectangle with the dimension 40 × 3 mm, as shown in Figure 1b. After cutting, we performed the hydrophilic treatment of OSTE pillar forest using oxygen plasma (at 1000 mTorr for 8 min, Plasma Cleaner, PDC-002, Harrick Plasma, Ithaca, NY, USA). Then, we assembled the microfluidic device using filtration membrane, OSTE pillar forest, and hydrophilic tape. In detail, at first, we used the hydrophilic tape to cover the surface of the rectangle plasma channel; then, we coated some glue on the edge of round part of OSTE pillar forest and glued the filtration membrane onto it, as shown in Figure 2a and Appendix A.2. The pore size on one side of the filtration membrane is bigger than that on the other side, as shown in Figure 2b. The side with smaller pores is in contact with OSTE pillar forest, while the other side is in the upside for sample loading. There was a wedge gap formed in the interface between hydrophilic tape, filtration membrane, and OSTE pillar forest, which can help to enhance the capillary action [4]. During the experiments, we attached the test strips on a glass slide using double-sided tape.

After the preparation of test strips, we dropped the whole blood sample onto the center of the filtration membrane by a pipette and used a digital camera (Canon EOS RP, Japan) to record the experimental video for data analyses. The data analyses were perforemd by a Video Analysis and Modeling Tool Tracker (https://physlets.org/tracker/, accessed on 18 January 2021). After the experiments, we cut the plasma channels off the test strips and used a mini centrifuge to spin out the plasma sample [17]. Then, we checked the purity of the plasma sample by microscope (Leica DM IL LED Fluo, Leica, Wetzlar, Germany) and measured the protein concentration in the plasma using a bicinchoninic acid (BCA) assay (BL521A, Biosharp Life Sciences, Hefei, China), of which the details can be found in Appendix A.3. We compared the protein concentration in separated plasma with that in the plasma got by centrifugation and calculated the protein recovery rate. For each blood sample, every experiment was repeated six times.

## 3. Results and Discussion

The head of the OSTE pillar is a rectangle, as shown in Figure 1c,d. For the dimensions of OSTE pillars, with *p* value as 350 μm, the average side length is 76 μm, the distance between pillars is 274 μm, and the depth of groove is 54 μm. With *p* value as 500 μm, the average side length is 120 μm, the distance between pillars is 380 μm, and the depth of groove is 32 μm. As the intensity distribution of the laser beam follows a Gaussian distribution, the profile of groove between OSTE pillars also resembles a Gaussian curve [35], as shown in Appendix A.4. We characterized the flow behavior and capillary flow rate of water on these two different substrates (more details can be found in Appendix A.5) and chose the OSTE pillar forest fabricated with *p* = 350 μm as the substrate material, which can provide a bigger volume capacity at 6.32 μL/cm${}^{2}$. The fabrication process of OSTE pillar forest is fast. All of the steps including pattern design, PDMS molding, OSTE molding, laser cutting, and hydrophilic treatments can be performed in less than three hours, which can benefit the iteration of device design.

After the preparation of the devices (as shown in Figure 2c), we loaded the whole blood sample on the center of filtration membrane. At first, the whole blood sample penetrated the filtration membrane; then, the separated plasma flowed out from the other side of the filtration membrane and formed a capillary bridge between the filtration membrane and OSTE pillar forest substrate. The OSTE pillar forest acted as a capillary pump to constantly inspire the plasma until the plasma channel was filled. During the experiments (as shown in Figure 2d), the plasma filled OSTE pillar forest below the filtration membrane at first and then the rectangle plasma channel. To find the suitable blood volume that the devices can process, we flowed blood samples with different volumes onto the filtration membrane. The amount of blood should be large enough to generate plasma to fill the plasma channel but cannot leave too much excess blood on the filtration membrane. We tested blood samples with different volumes from different pigs and chose to use 45 μL as the optimal sample volume. For the process rate of our devices, 45 μL of whole blood can be processed within 72 s, which is much higher than many passive devices for plasma separation [20,36,37,38]. We obtained the plasma separation yield by calculating the ratio of plasma separated to the total plasma in whole blood. We used Equation (1) to calculate the plasma separation yield:

(1) $$Separation\;yield=\frac{Volume_{separated\;plasma}}{Volume_{whole\;blood}\times \left(1-hematocrit\right)}.$$

Volume of separated plasma can be calculated by Equation (2):

(2) $$Volume_{separated\;plasma}=Volume\;capacity\times Area_{pillar\;forest}.$$

The average plasma separation yield of our devices is 60.0% ± 8.1%. We investigated the flow behavior of plasma on the plasma channel by studying the relation between pump distance and flow time. When the plasma reached the rectangle channel after filling the round region below the filtration membrane, the capillary pressure kept constant and the fluidic resistance increased with the capillary pumping, the distance–time curves should follow the Washburn Equation [39,40]: dx/dt ∼ t${}^{0.5}$, which is used to describe the behavior of capillary flow in capillary tubes or porous media (x indicates the penetrating distance, and t is the time). We used the Washburn Equation for fitting of the experimental data, and the details can be found in Reference [41]. The average R${}^{2}$ of the fitting for all of the tests is larger than 0.987.

**Figure 3:**
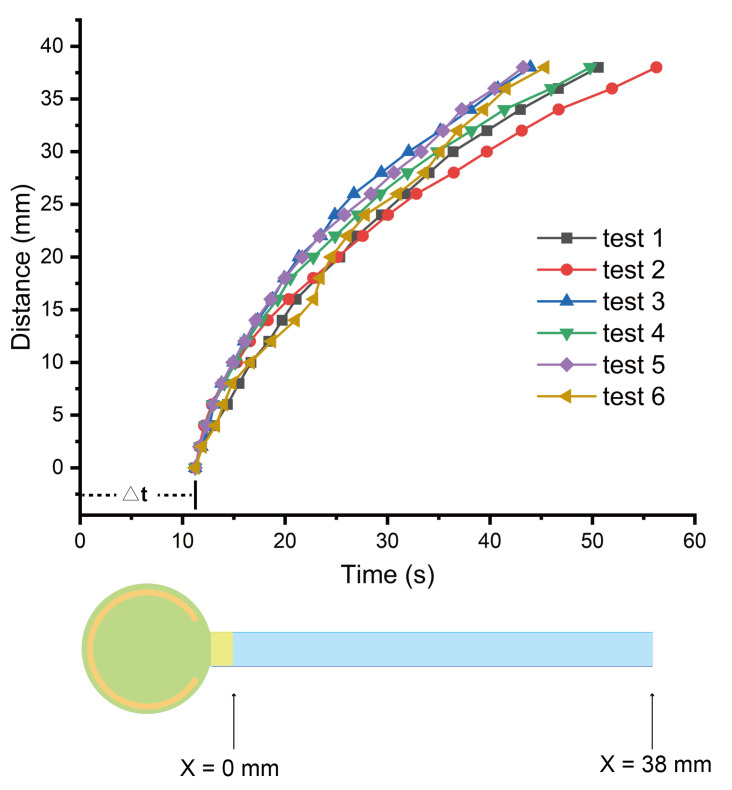
The flow behavior of the same blood sample on different test strips. We tested one blood sample (with hematocrit as 30.4%) on six different devices and obtained the distance–time curves of plasma flow. Δt indicates the time from loading the blood sample to when the plasma appears at distance X = 0 mm. The average Δt for this sample is 11.24 s.

**Figure 4:**
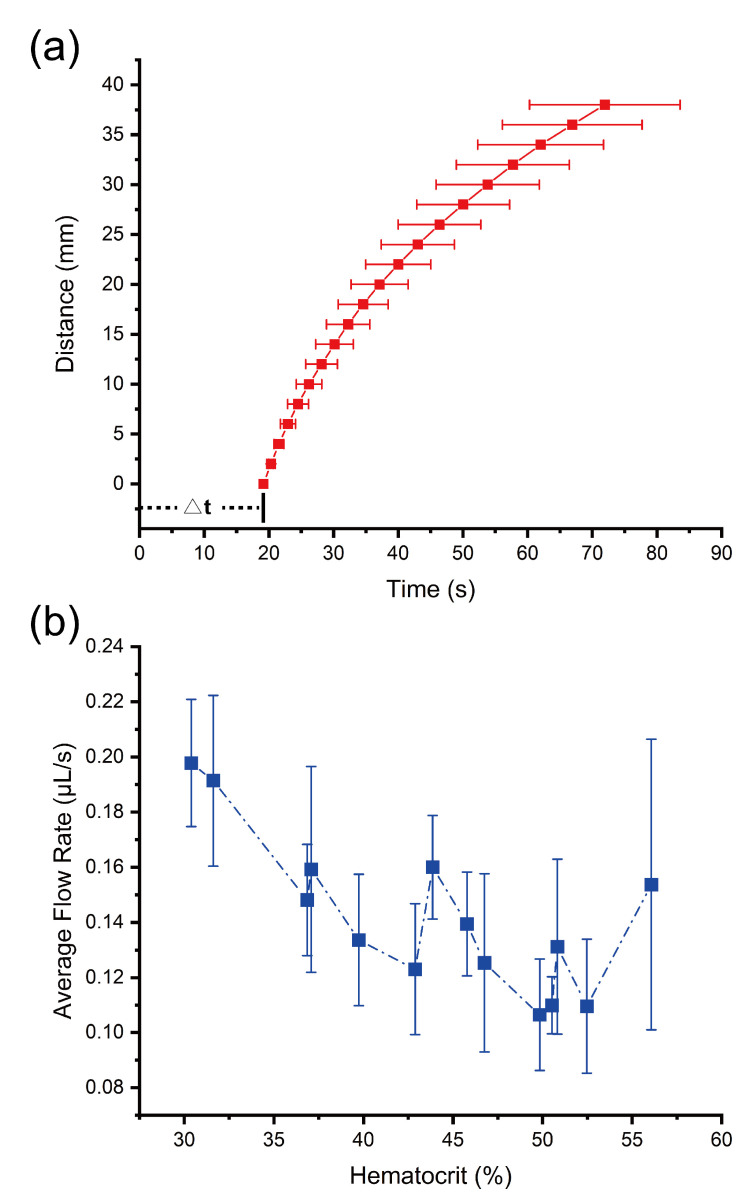
The flow behavior of different blood samples on the devices. We tested 14 blood samples on the devices, and these blood samples have hematocrit values from 30.4% to 56.1%. (**a**) The average distance–time curve of different blood samples: the average Δt for these 14 samples is 19.15 s. (**b**) The relation between hematocrit value and average flow rate.

We investigated the variation from device to device by comparing the flow behaviors of same blood sample (with hematocrit as 30.4%) on different devices. As shown in Figure 3, there are six curves of distance versus time, which are from one blood sample tested on six devices. As we can see from this figure, different curves are very similar. The average flow rate on different devices is 0.198 ± 0.023 μL/s, with a 11.6% variation. Furthermore, we investigated the variation from sample to sample by comparing the flow behavior of different blood samples (with hematocrit from 30.4% to 56.1%) on the devices. We tested 14 blood samples in total and obtained their average curves of distance versus time, shown in Figure 4a. The average flow rate of different blood samples is 0.142 ± 0.028 μL/s. Noticeably, there is a rough trend that the average flow rate of plasma decreased with the increase in hematocrit value, as shown in Figure 4b. A higher hematocrit could lead to a larger fluidic resistance and, therefore, a smaller flow rate. It is possible to further develop this device into a microfluidic device to measure hematocrit using the total flow time as the index [42].

We used Equation (3) to calculate the protein recovery rate of our devices:

(3) $$Protein\;recovery\;rate=\frac{Protein\;conc._{separated\;plasma}-Protein\;conc._{control}}{Protein\;conc._{centrifuged\;plasma}}.$$

$Protein\;conc._{separated\;plasma}$ refers to the protein concentration in the separated plasma by our devices, $Protein\;conc._{centrifuged\;plasma}$ refers to the protein concentration in the plasma obtained by centrifugation, and $Protein\;conc._{control}$ refers to the protein concentration in the separated sample when we load phosphate buffered saline (PBS) at the same volume onto our devices. We chose four samples to calculate the protein recovery rate and found that the protein recovery rate was 85.5%, which is similar to that in previous reports [15,17,43]. Furthermore, we checked the purity of separated plasma and found that there were few red blood cells in it, which indicated that the plasma separated by our devices had a high purity with a purification efficiency as 99.0% (see the details in Appendix A.3) [14].

We performed a comparison of our device with other passive separation devices in recent reports, as shown in Table 1. From this table, we can see that the overall performance of our device is on par with state-of-the-art technologies. Our device can process whole blood samples with a wider hematocrit value range in comparison with other devices. For separation yield and process rate, the performance of our device is above the medium level among the nine devices listed. The separation yield of our device ranks third, and the process rate ranks second among the devices of which relevant data are available. In terms of shelf life, our device has a shelf life of at least two months (details can be found in Appendix A.6). The cost of materials for a single device in this work is ∼0.23 USD (details in Appendix A.7), which fits well for point-of-care diagnostics.

Depending on specific applications, it is easy to scale up or down our device to fit the sample volume processed. Mainly, the sample volume that can be processed is limited by the area of the filtration membrane and the volume capacity of the substrate (OSTE pillar forest). According to information provided by the manufacturer of filtration membrane, the recommended blood volume is 35–45 μL/cm${}^{2}$. Therefore, the blood sample volume should be less than the capacity of the filtration membrane:

(4) $$Volume_{whole\;blood}<=45\;\mu {L/\mathrm{cm}}^{2}\times Area_{filtration\;membrane}.$$

Next, the area of pillar forest can be determined using Equations (1) and (2).

By improving the manufacturing method, it is possible to mass produce our devices. Injection molding can be used to fabricate OSTE pillar forest with a higher efficiency [25,27,44]. As different parts of our device are assembled by gluing or taping, commercial laminating technology is also applicable to the assembly of our device, which enables large-scale industrial manufacturing.

## 4. Conclusions

In summary, we developed a facile way to fabricate a substrate for capillary flow: OSTE pillar forest, and used it together with a filtration membrane to make a passive microfluidic device for plasma separation. Our device can process whole blood samples in a fast rate as 45 μL/72 s. It works for different blood samples with a hematocrit from 30.4% to 56.1%. The performance of our device on separation yield and protein recovery rate are also comparable with that in recent reports. Since our device separates the plasma from whole blood in a lateral flow test format, it can be immediately developed into a lateral flow test for biomarker detection in whole blood samples. The unique surface chemistry and optical properties of polymer OSTE make it easy to couple immunoassays on the OSTE pillar forest substrate.

## Figures and Tables

**Table 1:** Performance comparison between our device and other passive devices for plasma separation.

Reference	Methods	Hematocrit	Separation Yield	Protein Recovery Rate	Volume/Time (Process Rate)
Gao et al. [43]	Membrane filter	45%	71.70%	82.3%	60 μL/360 s
Samy et al. [45]	Wetting and Sedimentation	-	22–49%	-	-
Shamsi et al. [16]	Zweifach–Fung effect	-	66.6 %	-	-
Kuo et al. [46]	Fishbone filtration	45%	15%	-	10 μL/75 s
Baillargeon et al. [18]	Membrane filter	30%	53.8%	-	-
Liu et al. [47]	3D Parylene filter	-	42%	-	2000 μL/300 s
Son et al. [15]	Membrane filter	38%	20%	89%	-
Maria et al. [14]	Wetting and Sedimentation	45%	-	-	10 μL/900 s
Our device	Membrane filter	30.4–56.1%	60.0%	85.5%	45 μL/72 s

‘-’ means not provided.

**Figure A1:**
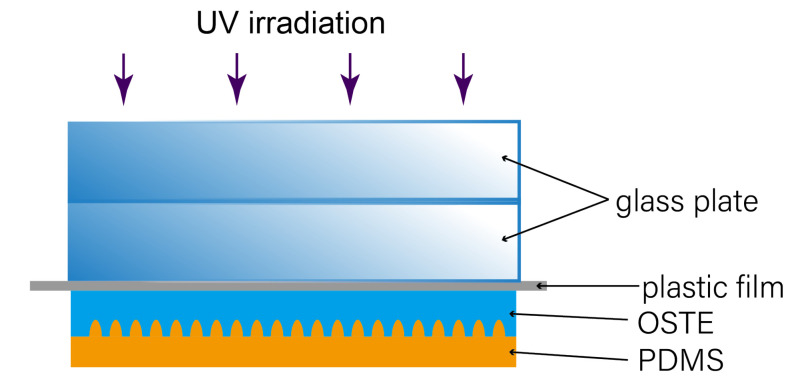
Fabrication setup of molding OSTE on PDMS.

**Figure A2:**
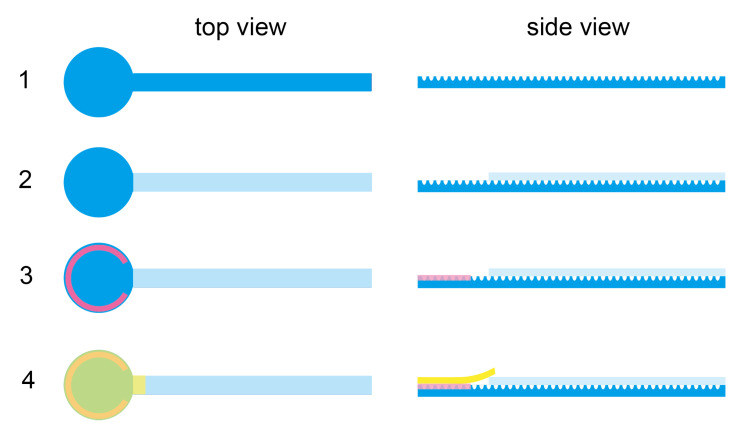
Assembly of the microfluidic device. The picture on the left shows the assembly steps from the top view, and the picture on the right shows the assembly steps from the side view.

**Figure A3:**
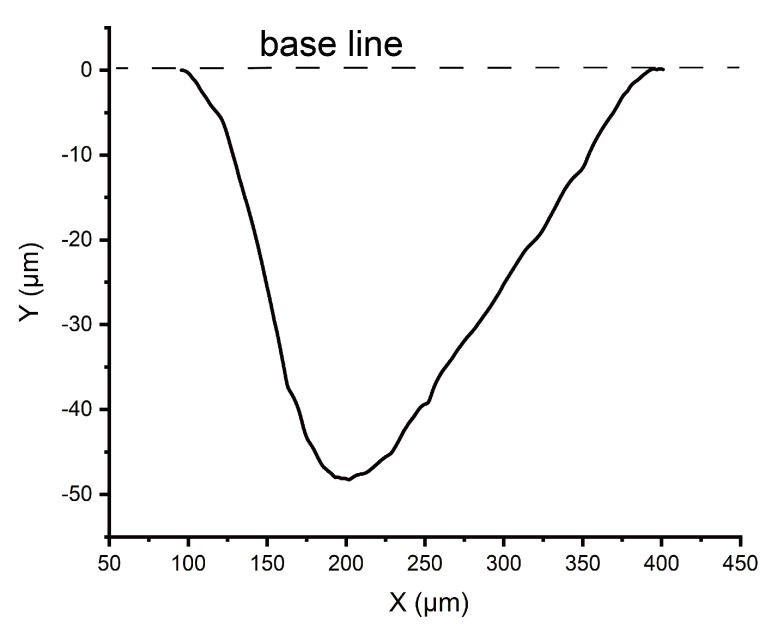
Profile of a micro groove on OSTE.

**Figure A4:**
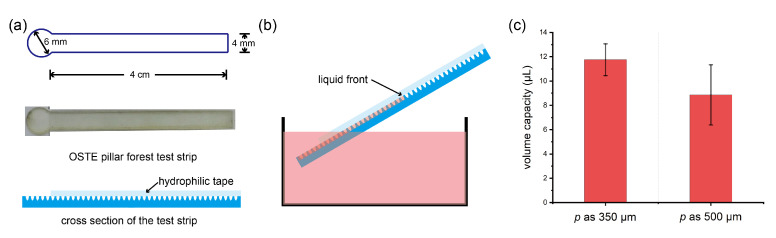
Water absorption experiments on the OSTE pillar forest substrate. (**a**) The design of test strips of OSTE pillar forest (up), the picture of a test strip (middle), and the schematic of the cross section of the test strip (below). (**b**) The schematic of the experimental setup. (**c**) The comparison of water volume absorbed on test strips with different *p* values.

**Figure A5:**
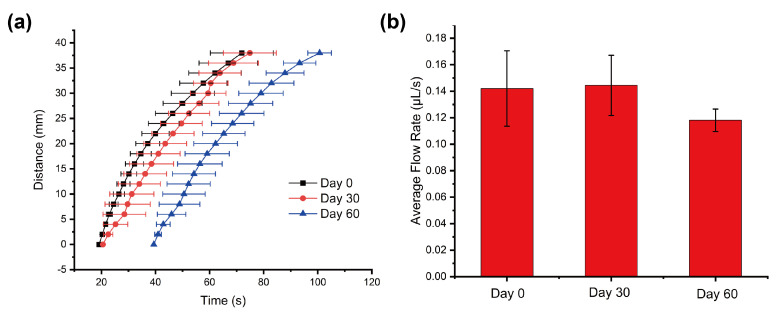
Flow behavior of whole blood samples on devices with shelf life of 0 day, 30 days, and 60 days. (**a**) A comparison of the distance-time curves and (**b**) a comparison of the average flow rates of plasma.

## Data Availability

The data are available upon request to guoweijin@stu.edu.cn.

## Appendix A

### Appendix A.1. Fabrication Setup of Molding OSTE on PDMS

As shown in Figure A1, when we molded OSTE on PDMS, we first poured OSTE on the PDMS mold and then we used a plastic film (3M 2910 Transparency Film, Maplewood, MN, USA) to cover OSTE. After that, we placed two thick glass plates (customized with dimension of 12.7 × 12.7 × 0.6 cm) on the plastic film to make sure the OSTE piece is flat. OSTE was cured by flood UV irradiation and removed from PDMS.

### Appendix A.2. Assembly of the Microfluidic Device for Plasma Separation

As shown in Figure A2, there were four steps of assembly for the microfluidic device: 1. we prepared the OSTE pillar forest, the hydrophilic tape, and the filtration membrane; 2. we covered the rectangle channel of OSTE pillar forest substrate with hydrophilic tape; 3. we coated the free nail glue on part of the edge of OSTE pillar forest; and 4. we glued the filtration membrane on OSTE pillar forest and slightly pressed the filtration membrane.

### Appendix A.3. Purity Check and Protein Concentration Measurement by BCA Assay

For the purity check of plasma separated by our devices, at first, we dropped 1 μL of the plasma sample on a glass slide (2.5 × 7.5 cm); then, we placed another glass slide (2.4 × 2.4 cm) on the plasma drop and let the plasma spread uniformly. Then, we checked if there was blood cells in the plasma sample by microscope (Leica DM IL LED Fluo, Leica, Germany). We used the equation below to calculate the purification efficiency [14]:

(A1) $$Purification\;Efficiency=(1-\frac{Number\;of\;cells\;in\;plasma\;collected\;by\;our\;devices}{Number\;of\;cells\;in\;whole\;blood\;samples})\times 100\%.$$

The number of cells in plasma collected by our devices and in whole blood samples refers to the number of cells in a field of view of 100 × 100 μm. In total, we tested nine samples, and each sample was tested three times. The purification efficiency of our devices is 99.0%.

There were seven steps of protein concentration measurement by BCA assay: 1. we prepared the bovine serum albumin (BSA) standard solution with nine concentrations including 0 μL/mL, 25 μL/mL, 125 μL/mL, 250 μL/mL, 500 μL/mL, 750 μL/mL, 1000 μL/mL, 1500 μL/mL, and 2000 μL/mL; 2. we took 2 μL centrifuged plasma or separated plasma and diluted it 100 times with 198 μL PBS; 3. we added 20 μL of the BSA standard solution, centrifuged plasma solution, separated plasma solution, and separated PBS to a 96-well dish, in which the separated PBS was from the control group where PBS was used as the sample liquid; 4. we prepared the BCA solution by mixing the BCA solution A and BCA solution B in a 50:1 ratio; 5. we added 200 μL of BCA solution to each well, mixed it thoroughly, and placed the dish in an incubator at 37 ${}^{\circ }$C for 30 min; 6. we took the 96-well dish out, placed it in a microplate reader to measure the light absorbance at 562 nm, and obtained the data; and 7. we plotted the standard curve and obtained the fitting equation to calculate the protein concentration of the samples according to the light absorbance value.

### Appendix A.4. Appendix Profile of Micro Grooves on OSTE

We use a step profiler (Dektak XT, Bruker, Billerica, MA, USA) to measure the surface profile of OSTE pillar forest, which shows that the profile of micro grooves on OSTE is similar to a Gaussian curve. An example of a groove profile is shown in Figure A3.

### Appendix A.5. Appendix Volume Capacity of Water Absorption on OSTE Pillar Forest

The purpose of this experiment was to determine the volume capacity of water absorption on the OSTE pillar forest substrate, which was covered by a piece of hydrophilic tap. The test strip in this experiment (as shown in Figure A4a) was slightly different from the device for plasma separation in shape and size. The experiment had four steps: 1. we measured the weight of an unused test strip; 2. the test strip was dipped into DI water, which was dyed red, as shown in Figure A4b, and when the liquid front reached the end of the test strip, it indicated that the strip was filled with water; 3. we measured the weight of the test strip and calculated the difference before and after water absorption; and 4. we calculated the volume of water absorbed according to weight difference and volume capacity per unit area by dividing the area of the test strip:

(A2) $$Volume\;capacity=\frac{Weight\;difference}{Density_{water}\times Area_{pillarforest}}.$$

For each type of test strip with different *p* values, the experiment was repeated three times. The result of the experiment is that the volume capacity of the test strip with *p* = 350 μm is 11.76 μL (6.32 μL/cm${}^{2}$) and that of test strip with *p* = 500 μm is 8.87 μL (4.76 μL/cm${}^{2}$), as shown in Figure A4c.

### Appendix A.6. Appendix Shelf Life of Our Devices

We tested the performance of our devices 30 days and 60 days after the device preparation. We flowed three different blood samples onto the devices, analyzed the distance–time curve, and calculated the average flow rates. We compared the performance of devices at day 0, day 30, and day 60. As shown in Figure A5, the time–distance curves at day 0 and day 30 were very similar, and the average flow rates were very close. The average flow rate at day 60 is slightly smaller than flow rates at days 0 and 30, and the waiting time is longer than that at day 0 and day 30. In short, there was no significant difference between the devices’ performance at day 0 and day 30, and we see obvious decay in performance at day 60.

Although some hydrophilic groups are generated on OSTE pillar forest, these groups may react with oxygen in the air when stored in air environment and, thereafter, affect the shelf life of the devices. However, if we can find a better method for packaging the devices such as in a nitrogen environment, we may be able to increase their shelf life.

### Appendix A.7. Appendix Estimation of Cost of Our Device

In Table A1, we performed an estimation of the cost of a single device in this work. We considered the materials including the hydrophilic tape, the blood filtration membrane, and the polymer OSTE. The cost per device is ∼0.23 USD, which indicates that our device is promising in applications of point-of-care diagnostics.

**Table A1:** Cost estimation of one device in this work.

Material	Unit cost	Material per Device	Cost per Device (USD)
Blood filtration membrane	0.000233 USD/mm${}^{2}$	165 mm${}^{2}$	0.0384
Hydrophilic tape	0.0000682 USD/mm${}^{2}$	120 mm${}^{2}$	0.00818
Free nail glue	0.0302 USD/mL	0.01 mL	0.000302
OSTE	0.127 USD/g	1.43 g	0.182
		Total:	0.229
